# Improving clinical utility of GAD65 autoantibodies by electrochemiluminescence assay and clinical phenotype when identifying autoimmune adult-onset diabetes

**DOI:** 10.1007/s00125-021-05492-6

**Published:** 2021-07-16

**Authors:** Yong Gu, Xiaofan Jia, Tanwi Vartak, Dongmei Miao, Fran Dong, Samuel T. Jerram, Marian Rewers, Assiamira Ferrara, Jean M. Lawrence, Liping Yu, R. David Leslie, the Action LADA consortium and the Diabetes in Young Adults (DiYA) Study Group, Richard David Leslie, Mohammed I Hawa, Paolo Pozzilli, Henning Beck-Nielsen, Knud Yderstraede, Steven Hunter, David Hadden, Raffaella Buzzetti, Werner Scherbaum, Hubert Kolb, Nanette C. Schloot, Jochen Seissler, Guntram Schernthaner, Jaakko Tuomilehto, Cinzia Sarti, Alberto De Leiva, Eulalia Brugues, Didac Mauricio, Charles Thivolet, Jean M Lawrence, Assiamira Ferrara, Jeff M. Slezak, Charles Quesenberry, Sharon Saydah, Liping Yu, Marian Rewers

**Affiliations:** 1 Barbara Davis Center for Diabetes, University of Colorado School of Medicine, Aurora, CO USA; 2 Endocrinology and Metabolism, First Affiliated Hospital of Nanjing Medical University, Nanjing, China; 3 Endocrinology, Beijing Hospital, National Center of Gerontology, Beijing, China; 4 Centre for Immunobiology, Blizard Institute, Barts and the London School of Medicine and Dentistry, Queen Mary University of London, London, UK; 5 Division of Research, Kaiser Permanente Northern California, Oakland, CA USA; 6 Department of Research & Evaluation, Kaiser Permanente Southern California, Pasadena, CA USA

**Keywords:** Adult-onset diabetes, Autoantibodies, Biomarker, ECL assay

## Abstract

**Aims/hypothesis:**

It is important to differentiate the two major phenotypes of adult-onset diabetes, autoimmune type 1 diabetes and non-autoimmune type 2 diabetes, especially as type 1 diabetes presents in adulthood. Serum GAD65 autoantibodies (GADA) are the most sensitive biomarker for adult-onset autoimmune type 1 diabetes, but the clinical value of GADA by current standard radiobinding assays (RBA) remains questionable. The present study focused on the clinical utility of GADA differentiated by a new electrochemiluminescence (ECL) assay in patients with adult-onset diabetes.

**Methods:**

Two cohorts were analysed including 771 diabetic participants, 30–70 years old, from the Action LADA study (*n* = 6156), and 2063 diabetic participants, 20–45 years old, from the Diabetes in Young Adults (DiYA) study. Clinical characteristics of participants, including requirement of early insulin treatment, BMI and development of multiple islet autoantibodies, were analysed according to the status of RBA-GADA and ECL-GADA, respectively, and compared between these two assays.

**Results:**

GADA was the most prevalent and predominant autoantibody, >90% in both cohorts. GADA positivity by either RBA or ECL assay significantly discriminated clinical type 1 from type 2 diabetes. However, in both cohorts, participants with ECL-GADA positivity were more likely to require early insulin treatment, have multiple islet autoantibodies, and be less overweight (for all *p* < 0.0001). However, clinical phenotype, age at diagnosis and BMI independently improved positive predictive value (PPV) for the requirement of insulin treatment, even augmenting ECL-GADA. Participants with GADA detectable by RBA, but not confirmed by ECL, had a phenotype more similar to type 2 diabetes. These RBA-GADA positive individuals had lower affinity GADA compared with participants in which GADA was confirmed by ECL assay.

**Conclusions/interpretation:**

Detection of GADA by ECL assay, given technical advantages over RBA-GADA, identified adult-onset diabetes patients at higher risk of requiring early insulin treatment, as did clinical phenotype, together allowing for more accurate clinical diagnosis and management.

**Graphical abstract:**

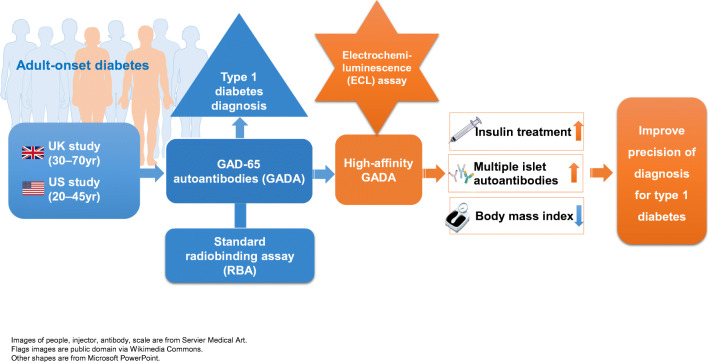

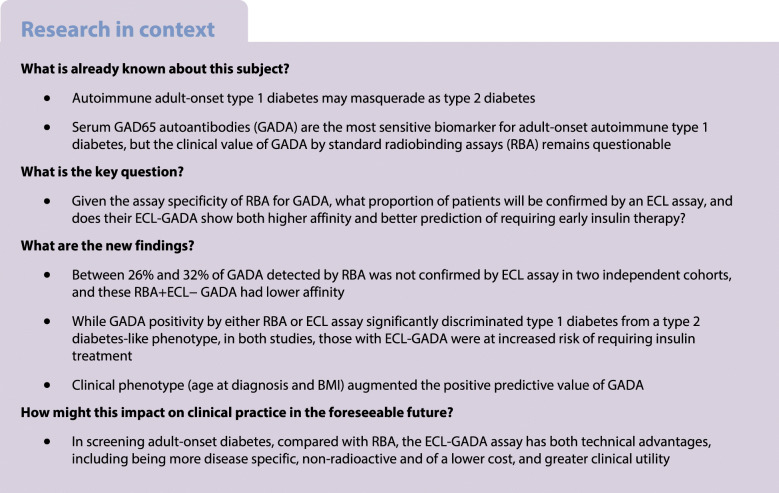



## Introduction

Type 1 diabetes has been recently shown in epidemiological studies to be predominantly a disease of adult onset [1–3]. Adult-onset type 1 diabetes is more heterogeneous than childhood-onset type 1 diabetes; the majority of adults do not require insulin treatment initially and have been arbitrarily defined as having latent autoimmune diabetes in adults (LADA) when off insulin treatment at least 6 months post-diagnosis [4]. Distinguishing adult-onset type 1 diabetes from type 2 diabetes is clinically important and commonly employs the GAD65 autoantibody (GADA) assay [5]. Since GADA is the dominant, often the only, diabetes-associated autoantibody in adult-onset diabetes, it is possible that some GADA positive participants have either biologically false positive GADA or GADA of limited clinical utility. Following extensive validation, our recently developed electrochemiluminescence (ECL) assay for GADA was more predictive of progression to type 1 diabetes than current radiobinding assays (RBA) in several major clinical trials [6, 7]. ECL assays discriminate individuals with high affinity GADA at high disease-risk from those identified by RBA with low affinity GADA at low disease-risk. The present study aims to evaluate, for the first time, GADA by ECL assay, compared with the standard RBA, in individuals with adult-onset diabetes. We hypothesised that identification of GADA using the ECL assay, compared with the RBA, would have greater clinical utility in screening adult-onset autoimmune diabetes by allowing for more accurate clinical diagnosis to the benefit of clinical care.

## Methods

### Participants and samples

**Fig. 1:**
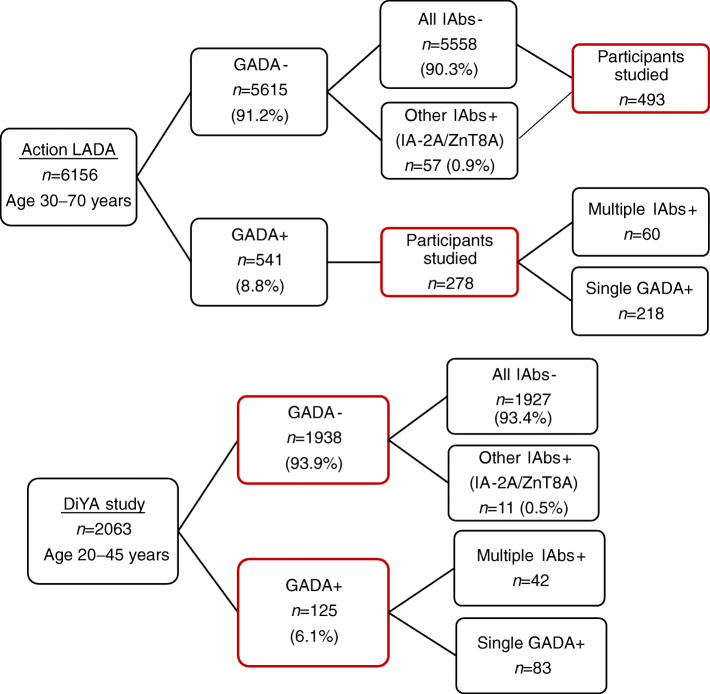
Participants studied from the Action LADA and DiYA studies

Serum samples were obtained from two large clinical studies, the Action LADA study in the UK and the Diabetes in Young Adults (DiYA) study in the USA. The Action LADA study is a cross-sectional study of adult participants aged 30 to 70 years, with recent-onset diabetes diagnosed within 5 years (originally *n* = 6156) (www.actionlada.org) [5]. For the current study, 771 participants with sufficient available sera were selected, including adult-onset diabetes patients positive for RBA-GADA (*n* = 278, 51.4% of the Action LADA cohort with RBA-GADA) with or without insulinoma-associated antigen-2 autoantibodies (IA-2A) or zinc transporter-8 autoantibodies (ZnT8A), and patients negative for RBA-GADA (*n* = 493) (see Fig. 1). The mean (SD) age was 51.5 (7.6) years, 54.0% were male, 69.5% were non-Hispanic White and mean duration of diabetes was 2.4 (4.2) years with similar age (+/−5%) and diabetes duration (+/−5%) between the two groups. DiYA is a prospective study to estimate the incidence of type 1 diabetes among younger adults, aged 20 to 45 years, with recent-onset diabetes [8]. Samples were collected among participants (mean [SD] age 36.9 [6.1] years at diagnosis, 46% male, 24% non-Hispanic White) at a median diabetes duration of 10 months. All 2063 DiYA participants whose samples were measured for islet autoantibodies (IAbs) by RBA were included in the study, including individuals (*n* = 125) positive for RBA-GADA with or without IA-2A or ZnT8A, and participants (*n* = 1938) negative for RBA-GADA (Fig. 1). Clinical information such as age, sex, ethnicity, BMI and insulin treatment were collected from both studies. In the Action LADA cohort, information for insulin treatment was available for 694 (90%) of participants. In both studies, individuals on insulin treatment at the latest sample and within 5 years of diagnosis were considered to have ‘early insulin therapy’. Both studies were approved by the responsible local ethics committees (institutional review boards) and the study participants gave informed consent.

### RBA for GADA, IA-2A and ZnT8A

RBA for all three autoantibodies (GADA, IA-2A and ZnT8A) were performed at the Blizard Institute, London, for the Action LADA study, and at the Barbara Davis Center for Diabetes, University of Colorado, USA, for the DiYA study. The methodologies have been described previously [5, 6], and both laboratories used full length GAD65 in their RBA. The GADA cut-offs were 68 (index) for the Blizard Institute laboratory and 20 (DK units) for the Barbara Davis Center for Diabetes laboratory. GADA achieved adjusted sensitivities of 64% at 95% specificity at the Blizard Institute laboratory in the Islet Autoantibody Standardization Program (IASP) workshop of 2012 when the RBA were performed for the Action LADA study, and sensitivity of 82% at 99% specificity at the laboratory in the Barbara Davis Center for Diabetes (lab ID: 133) during the IASP workshop of 2018 [9] when the RBA were performed for the DiYA study.

### ECL assay for GADA

ECL-GADA assays were measured at the Barbara Davis Center for Diabetes laboratory as previously described [6]. In brief, serum samples were mixed with both sulfo-tag- and biotin-labelled GAD65 (full length) antigen proteins for overnight incubation at 4°C. The antigen–antibody complexes labelled with biotin were captured by a streptavidin-coated plate, and the sulfo-tag labelled complexes gave the signals with ECL. The results were expressed as an index against internal standard GAD65 positive controls. The ECL assay cut-off index of 0.023 for GADA was set at the 99th percentile over 100 healthy controls, and the ECL inter-assay CV was 8.8% (*n* = 10) for GADA. In the 2018 IASP Workshop [9], sensitivity for ECL-GADA was 82% and specificity was 99%.

### GADA affinity assay

The GADA affinity assay was carried out as previously described [6], and the native GAD65 protein (Diamyd, Pittsburgh, PA, USA) was used in an RBA-GADA competitive assay. For the present study, 43 samples in total were analysed, including 17 samples from Action LADA (9 GADA positive in both RBA and ECL assay formats, and 8 GADA positive in RBA alone) and 26 samples from DiYA (18 GADA positive in both RBA and ECL assay formats, and 8 GADA positive in RBA alone). Two concentrations of unlabelled GAD65 protein (4.6 × 10^−10^ mol/l and 4.6 × 10^−9^ mol/l) were used for the competition assay. Inhibitions for 50% of signals at the concentrations of unlabelled GADA were compared between antibodies.

### Statistical analysis

All statistical analyses were performed using GraphPad Prism v9.1.0 Software (https://www.graphpad.com/scientific-software/prism/) and SAS v9.4 (SAS Institute, Cary, NC, USA; https://support.sas.com/downloads/package.htm?pid=2490). Continuous variables are reported as mean or median and categorical variables are reported as the number and percentage of participants with the characteristic of interest and ORs (95% CI). Pearson’s correlation analysis was performed to assess the strength of correlation between levels of GADA in the two assays. To compare the levels of BMI across the four diabetes-associated autoantibody groups, ANOVA was used. Logistic regression analyses were used to test for difference in per cent of requirement for insulin treatment and per cent of multiple IAbs across the four groups. Firth’s penalised likelihood approach was used to minimise the analytical bias caused by small samples, rare events and incomplete separation. Sensitivity and positive predictive value (PPV) for the prediction of early insulin use were calculated for various cut-offs. For all tests, *p* values <0.05 were considered significant with a two-tailed test.

## Results

### In adult-onset autoimmune diabetes GADA is dominant

Two distinct cohorts covering a wide age range of recently diagnosed adult-onset diabetes patients (Action LADA and DiYA) were studied using two different RBA-GADA assays, as well as assays for IA-2A or ZnT8A, in their respective centralised laboratories, while the same ECL- GADA assay was used for both cohorts in one centralised laboratory. Overall, IAb positivity was 9.7% (598/6156) in the Action LADA study and 6.6% (136/2063) in the DiYA study. RBA-GADA was the most prevalent autoantibody and predominated, 90.5% (541/598) in the Action LADA study and 91.9% (125/136) in the DiYA study, respectively, while other IAbs (IA-2A or ZnT8A) were detected in only a small fraction. In the total unselected Action LADA cohort (*n* = 6156), only 0.9% (57 of 6156) had IA-2A or ZnT8A alone [5]. In the DiYA cohort (*n* = 2063), only 0.5% (11/2063) had IA-2A or ZnT8 alone.

**Fig. 2:**
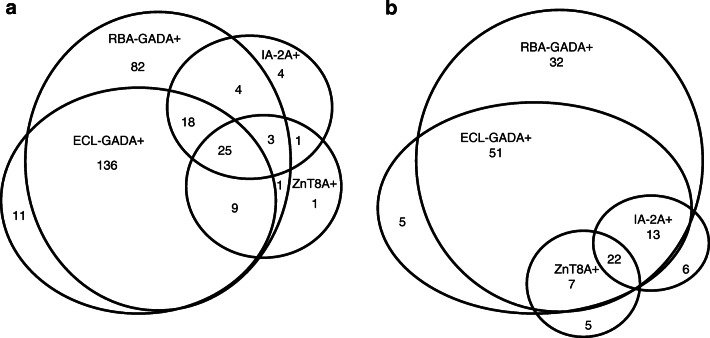
Overall IAb positivity including GADA, IA-2A and ZnT8A for the participants studied in the Action LADA study (**a**) and the DiYA study (**b**)

Unlike childhood-onset diabetes, the majority of adult-onset diabetes patients positive for IAbs in these studies were single positive for GADA without other IAbs (78.4% [218/278] of the Action LADA participants studied had single GADA+ and 66.4% [83/125] of the DiYA study participants had single GADA+). The overall IAb positivity, including GADA, IA-2A and ZnT8A is summarised in Fig. 2 for both the Action LADA (Fig. 2a) and the DiYA studies (Fig. 2b).

### Detection of GADA by either RBA or ECL assay has significant clinical utility to differentiate type 1 diabetes from type 2 diabetes for adult-onset diabetes

**Fig. 3:**
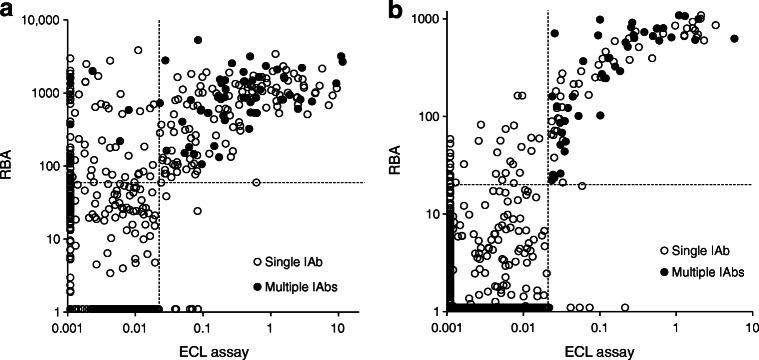
Comparison of GADA levels between RBA and ECL assay in the (**a**) Action LADA study (*n* = 771) and (**b**) DiYA study (*n* = 2063). The solid dots represent individuals positive for IA-2A and/or ZnT8A and the open dots negative for IA-2A and ZnT8A. The dotted lines indicate assay cut-offs. RBA-GADA was tested at the Blizard Institute laboratory, London, for the Action LADA study, with a cut-off value of 68 (index), and at the Barbara Davis Center for Diabetes laboratory, USA, for the DiYA study, with a cut-off value of 20 (DK units). The ECL-GADA assay was performed at the Barbara Davis Center for Diabetes for both Action LADA and DiYA studies, with a cut-off value of 0.023 (index)

Participants who were GADA positive in both assays, compared with individuals who were negative for GADA with both assays (presumptive type 2 diabetes, *n* = 482 in the Action LADA study and *n* = 1921 in the DiYA study), were: (1) leaner (*p* < 0.0001 and also for each assay considered separately); (2) more frequently positive for other IAbs i.e. IA-2A and/or ZnT8A (*p* < 0.0001 and also for each assay considered separately); and (3) more often required early insulin treatment (*p* < 0.0001 and also for each assay considered separately). In the Action LADA study, the risk of insulin therapy was significantly higher in participants who were GADA positive with both assays (OR 10.87 [95% CI 6.42, 18.39]; *p* < 0.0001), compared with participants who were negative for GADA with both assays, but also with either RBA alone (OR 6.51 [95% CI 4.02, 10.54]; *p* < 0.0001; PPV 28.6%) or ECL assay alone (OR 8.78 [95% CI 5.49, 14.03]; *p* < 0.0001; PPV 37.4%). The DiYA study also found the risk of requiring insulin therapy was significantly higher in individuals with both RBA-GADA and ECL-GADA positivity (OR 3.75 [95% CI 2.44, 5.75]; *p* < 0.0001), compared with participants negative for GADA with both assays, and also higher in those with either RBA-GADA alone (OR 2.61 [95% CI 1.77, 3.85]; *p* < 0.0001; PPV 34.4%) or ECL-GADA alone (OR 3.45 [95% CI 2.27, 5.26]; *p* < 0.0001; PPV 40.8%). Levels of GADA from RBA and ECL assays in both the Action LADA (*n* = 771) and DiYA (*n* = 2063) studies, are plotted in Fig. 3a and b, respectively. Levels in individuals who were GADA positive in both assays were correlated (for Action LADA r = 0.3208, *p* < 0.0001, and for DiYA r = 0.5771, *p* < 0.0001). Interestingly, in both cohorts, the risk of requiring early insulin therapy was not associated with levels of either RBA-GADA or ECL-GADA.

### RBA detected more GADA but was less specific than ECL assay

**Fig. 4:**
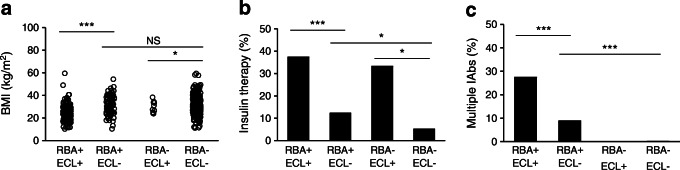
Clinical features were compared in four groups of participants with adult-onset diabetes from the Action LADA study: RBA-GADA+ confirmed by ECL assay, RBA-GADA+ not confirmed by ECL assay, ECL-GADA+ only, and GADA– in both RBA and ECL assay. (**a**) BMI values; (**b**) per cent of participants requiring early insulin treatment; (**c**) per cent of participants positive for multiple IAbs. *p < 0.05, ***p < 0.001 by ANOVA test or logistic regression analyses

**Fig. 5:**
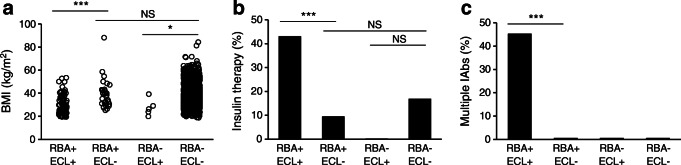
Clinical features were compared in four groups of participants with adult-onset diabetes from the DiYA study: RBA-GADA+ confirmed by ECL assay, RBA-GADA+ not confirmed by ECL assay, ECL-GADA+ only, and GADA– by both RBA and ECL assay. (**a**) BMI values; (**b**) per cent of participants requiring early insulin treatment; (**c**) per cent of participants positive for multiple IAbs. *p < 0.05, ***p < 0.001 by ANOVA test or logistic regression analyses

In both studies, more participants were identified as RBA-GADA positive than ECL-GADA positive. In the present Action LADA cohort (*n* = 771), 278 individuals were RBA-GADA positive by selection. In this cohort, 188/771 (24.4%) were positive for both RBA-GADA and ECL-GADA, 90 (11.7%) had RBA-GADA alone, while only 11 (1.4%) had ECL-GADA alone. In the DiYA cohort, 93/2063 (4.5%) had both RBA-GADA and ECL-GADA, 32 (1.6%) were positive for RBA-GADA alone, while only 5 (0.2%) had ECL-GADA alone. As such, between 26% (DiYA study) and 32% (Action LADA) of GADA detected by RBA was not detected by ECL. In both studies, ECL-GADA positivity was particularly reduced in individuals with single IAb positivity or RBA-GADA positivity alone. In the Action LADA cohort, ECL-GADA was only found in 62% of participants (136/218) with RBA-GADA alone, compared with participants with more than one autoantibody, i.e. GADA plus IA-2A and/or ZnT8A (52/60 [87%], *p* = 0.0003), as shown in Fig. 3a. Similarly in the DiYA study, ECL-GADA positivity was only found in 61% of individuals with RBA-GADA alone (51/83) and in 100% of participants with RBA-GADA and IA-2A and/or ZnT8A (42/42, *p* < 0.0001) as shown in Fig. 3b. Remarkably, participants defined by RBA-GADA positivity, but not confirmed by the ECL-GADA assay, had a phenotype more similar to type 2 diabetes from both the Action LADA and the DiYA cohorts as shown in Figs 4 and 5. They were more obese (mean BMI 30.7 kg/m^2^ vs 23.3 kg/m^2^ in the Action LADA cohort, 38.8 kg/m^2^ vs 29.5 kg/m^2^ in the DiYA cohort, both *p* < 0.0001), less likely to have multiple IAbs (8/90 [8.9%] vs 52/188 [27.7%], *p* = 0.0003) and 0/32 vs 42/93 [45.2%], *p* < 0.0001, respectively), and less often required early insulin treatment(10/81 [12.3%] vs 61/162 [37.7%] and 3/32 [9.4%] vs 40/93 [43.0%], respectively, both *p* < 0.0001).

Individuals who were RBA-GADA positive not confirmed by ECL assay, compared with individuals GADA negative in both assays, still showed increased requirement for early insulin treatment in the Action LADA cohort (Fig. 4b; 12.3% vs 5.2%), while it was the opposite in the DiYA cohort (Fig. 5b; 9.4% vs 16.8%). Levels of RBA-GADA in both cohorts were significantly lower in those who were ECL-GADA negative (*p* = 0.0002 for Action LADA and *p* < 0.0001 for DiYA), but absolute levels were not discriminatory (Fig. 3). Interestingly, in both studies, other IAbs (IA-2A and/or ZnT8A) did not increase the risk of requiring early insulin treatment in participants who were ECL-GADA positive. By contrast, in young adult-onset diabetes (DiYA), in individuals who were RBA-GADA positive, the addition of other IAbs was associated with increased frequency of early insulin treatment (21/42 [50%] vs 22/83 [26.5%], *p* = 0.01), but not in the older adult-onset diabetes (Action LADA).

### RBA-GADA not confirmed by ECL assay were of low affinity

**Fig. 6:**
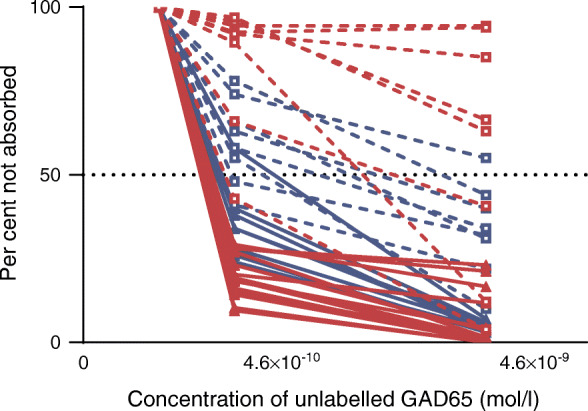
GADA affinity analysis. In total, 43 serum samples positive for RBA-GADA, including 17 samples from the Action LADA study (blue; nine confirmed by ECL assay and eight not confirmed by ECL assay) and 26 samples from the DiYA study (red; 18 confirmed by ECL assay and eight not confirmed by ECL assay) were analysed using competitive RBA and incubation with different concentrations of native GAD65 protein. GADA not confirmed by ECL assay (dotted line), compared with GADA confirmed by ECL assay (solid line), required higher concentrations of GAD65 protein for 50% maximal inhibition. Results were expressed as per cent of signal not absorbed

To confirm our previous finding that GADA affinity is higher when positive in the ECL-GADA assay as compared with RBA-GADA alone [6, 7], GADA affinity was analysed by competitive RBA in a total of 43 samples, including 17 samples from the Action LADA study (nine GADA positive in both RBA and ECL and eight only positive for RBA-GADA) and 26 samples from the DiYA study (18 GADA positive in both RBA and ECL, and eight samples only positive for RBA-GADA). The levels of RBA-GADA were selected to be similar between the two subgroups for affinity comparison. At the concentrations of 4.6 × 10^−10^ mol/l and 4.6 × 10^−9^ mol/l of unlabelled GAD65, GADA confirmed by ECL assay were absorbed by a mean of 76% and 94%, respectively, while GADA not confirmed by ECL assay were absorbed by a mean of 28% and 54%, respectively. Results (Fig. 6) confirmed that samples which were positive for RBA-GADA alone required a higher concentration of native GAD65 protein for 50% binding inhibition.

### Clinical phenotype augments GADA to predict early insulin use

Patient demographics impacted the risk of requiring early insulin treatment independently of GADA (Table 1). An earlier age at diagnosis (DiYA compared with Action LADA) and lower BMI (<25 kg/m^2^) were independently associated with higher PPV and together augmented the PPV of both RBA-GADA and ECL-GADA for early insulin treatment in both cohorts. RBA-GADA positivity and BMI <25 kg/m^2^ together increased PPV from 28.1% to 42.3% in the Action LADA cohort, and from 34.4% to 58.3% in the DiYA cohort. ECL-GADA positivity and BMI <25 kg/m^2^ together increased PPV from 37.4% to 45.7% in the Action LADA cohort, and from 40.8% to 55.3% in the DiYA cohort. ECL-GADA positivity and BMI <25 kg/m^2^ together had a higher PPV in the DiYA cohort than the Action LADA cohort (55.3% vs 45.7%) but at the cost of predictive sensitivity in the DiYA cohort (sensitivity only 5.7%) (Table 1).

**Table 1:** Clinical utility of GADA (RBA and ECL) assays and demographics (BMI) for predicting early insulin treatment in two cohorts (Action LADA and DiYA). PPV and sensitivity as a marker of early insulin treatment. Whilst PPV with ECL-GADA is higher than with RBA-GADA it can be further enhanced in both cohorts using low BMI (<25 kg/m^2^)

	Action LADA		DiYA	
Characteristic	PPV	Sensitivity	PPV	Sensitivity
BMI <25 kg/m^2^, *n* (%)	55/166 (33.1)	55/94 (58.5)	39/127 (30.7)	39/368 (10.6)
RBA-GADA+, *n* (%)	70/249 (28.1)	70/96 (72.9)	43/125 (34.4)	43/368 (11.7)
ECL-GADA+, *n* (%)	64/171 (37.4)	64/96 (66.7)	40/98 (40.8)	40/ 368 (10.9)
RBA-GADA+ & BMI < 25 kg/m^2^, *n* (%)	47/111 (42.3)	47/93 (50.5)	21/36 (58.3)	21/368 (5.7)
ECL-GADA+ & BMI < 25 kg/m^2^, *n* (%)	43/94 (45.7)	43/93 (46.2)	21/38 (55.3)	21/368 (5.7)

## Discussion

This study demonstrated that GADA, whether detected by RBA or ECL assays, recognised a clinical phenotype distinct from type 2 diabetes in adult-onset diabetes patients, in that individuals were leaner, with a higher frequency of multiple autoantibodies and earlier need for insulin therapy. This present investigation of two independent studies of an older and younger adult cohort in the UK and USA, respectively, covered a wide age range of adults in two different populations. In both cohorts, GADA was shown to be prevalent and dominant, the majority of individuals having only single GADA positivity without other IAbs (78% in the Action LADA study and 66% in the DiYA study). Of participants with GADA alone by RBA, 62% in the Action LADA cohort and 61% in the DiYA cohort were confirmed positive using ECL-GADA. In our previous studies of type 1 diabetes-risk screening, we repeatedly found that when screening either individuals from the general population (with type 1 diabetes-associated HLA alleles) or their relatives, of those who were RBA-GADA positive alone, 40% or fewer showed positivity confirmed by the ECL assay (data from three large clinical trials; Diabetes Autoimmunity Study in the Young [DAISY] [6], TrialNet [7] and The Environmental Determinants of Diabetes in the Young [TEDDY; unpublished data]). In an ongoing large screening study of unselected children from the general population (Autoimmunity Screening for Kids [ASK]), of those children with RBA-GADA positivity alone, only 20% were confirmed by ECL assay (L. Yu, unpublished data). Samples which were GADA positive by RBA alone, not confirmed by ECL assay, had lower affinity in both our previous studies, as well as this present study.

Higher affinity GADA predicts progression to type 1 diabetes [6, 7, 10], and in LADA predicts requirement of early insulin treatment [11]. We, and others, recently reported that RBA-GADA to N-terminal GAD epitopes contribute to low affinity positivity, while RBA-GADA assays using N-terminally truncated GAD improves clinical phenotyping in both type 1 diabetes and adult-onset diabetes to a comparable degree [12, 13]. In the TrialNet study, participants who were GADA positive by RBA, but not by ECL, did not show impaired glycaemia during follow-up (median 4.7 years), while ECL-GADA positive participants had comparable dysglycaemia to individuals with multiple IAbs at high risk of type 1 diabetes [14]. Our results likely reflect the known importance of enrichment by ascertainment to enhance the clinical utility of an assay, thereby explaining the striking variation in confirmation by ECL-GADA of positivity in individuals with RBA-GADA positivity alone, ranging from 20% (children in the general population), 40% (relatives of individuals with type 1 diabetes), compared here with 60% in adult-onset diabetes patients. While higher affinity GADA predicts progression to type 1 diabetes [6, 7, 10], and in both adult cohorts reported here, an increased requirement for insulin treatment [11], measurement of GADA affinity is time consuming and expensive with limited clinical applicability. Large prospective studies could establish whether different assays, seeking different epitope specificities or affinities, might benefit from clinical and predictive utility without losing predictive sensitivity.

Importantly, our data indicated that clinical phenotype, namely age at diagnosis and BMI, can also influence risk of progression to early insulin treatment independently of immunotype. Participants in the younger DiYA cohort consistently had higher PPV for GADA alone or in association with low BMI than the Action LADA cohort did, irrespective of the nature of the GADA assay. For the younger DiYA cohort, a low BMI had poor sensitivity as so few of them were lean. Nevertheless, our data implies an important role for features other than immunotype in predicting early insulin treatment, a novel observation that deserves further exploration.

There are some limitations to this study. The samples analysed from the initial Action LADA cohort are relatively few, albeit randomly selected based on sample availability from an initial cohort of over 6000 patients. Insulin therapy was started as a clinical decision and not based on a universal algorithm. The data are cross-sectional, though we predict that follow-up would likely increase the clinical utility of ECL-GADA [13, 14].

Importantly, both assay formats, including here the ECL assay, as well as epitope specificity with truncated GAD65 in RBA will likely be of practical clinical value in identifying autoantibodies predictive of early insulin therapy. Both assay formats improved the odds of requiring insulin therapy in the same LADA cohort as compared with the standard RBA-GADA assay. The ECL assay may be additionally valuable because it does not use radioactivity, is approved to equip other IAb (insulin autoantibodies, IA-2A and ZnT8A) assays with high affinity specificity with the same format, and is capable of large throughput with low cost, especially with its unique advantage of multiplexing to combine multiple autoantibody assays in one single well [15]. Once these results are confirmed, routine screening for GADA, such as the ECL-GADA assay, added to demographic features, should be explored further in patients with adult-onset diabetes for enhanced clinical utility.

## Data Availability

The datasets generated and/or analysed during the current study are available from the corresponding author on reasonable request.

## Appendix

**Members of the Action LADA Group:**

Richard David Leslie and Mohammed I Hawa (deceased), Blizard Institute, Queen Mary University of London, London, UK.

Paolo Pozzilli, University Campus Bio-Medico, Rome, Italy.

Henning Beck-Nielsen and Knud Yderstraede, University Hospital of Odense, Odense, Denmark.

Steven Hunter and David Hadden (deceased), Royal Victoria Hospital, Belfast, UK.

Raffaella Buzzetti, University La Sapienza, University of Rome, Italy.

Werner Scherbaum and Hubert Kolb, University of Düsseldorf, Düsseldorf, Germany.

Nanette C. Schloot, German Diabetes Centre, University of Düsseldorf, Düsseldorf and Clinic for Metabolic Diseases at University Hospital Düsseldorf. N.C. Schloot is guest scientist at German Diabetes Center, Institute of Clinical Diabetology, Düsseldorf and currently employed by Lilly Deutschland, Bad Homburg, Germany.

Jochen Seissler, Ludwig-Maximilians-University, Munich, Germany.

Guntram Schernthaner MD, Rudolfstiftung Hospital, Vienna, Austria.

Jaakko Tuomilehto and Cinzia Sarti, National Institute for Health and Welfare, Helsinki, Finland.

Alberto De Leiva and Eulalia Brugues, Universitat Autonoma de Barcelona, Barcelona, Spain.

Didac Mauricio, Hospital de la Santa Creu i Sant Pau, Barcelona, Spain.

Charles Thivolet, Hopital Edouard Herriot, Lyon, France.

**Members of the DiYA Study Group:**

Jean M Lawrence, Kaiser Permanente Southern California, Pasadena, CA, USA.

Assiamira Ferrara, Kaiser Permanente Northern California, Oakland, CA, USA.

Jeff M. Slezak, Kaiser Permanente Southern California, Pasadena, CA, USA.

Charles Quesenberry, Kaiser Permanente Northern California, Oakland, CA, USA.

Sharon Saydah, Centers for Disease Control and Prevention, Atlanta, GA, USA.

Liping Yu, Barbara Davis Center for Diabetes University of Colorado, Aurora, CO, USA.

Marian Rewers, Barbara Davis Center for Diabetes University of Colorado, Aurora, CO, USA.

## Abbreviations

DiYA: Diabetes in Young Adults
ECL: Electrochemiluminescence
GADA: GAD65 autoantibodies
IA-2A: Insulinoma-associated-2 autoantibodies
IAbs: Islet autoantibodies
IASP: Islet autoantibody standardization program
LADA: Latent autoimmune diabetes in adults
PPV: Positive predictive value
RBA: Radiobinding assays
ZnT8A: Zinc transporter-8 autoantibodies
